# CT-guided core needle biopsy of focal pulmonary lesions with coexisting interstitial lung abnormalities: a case-control study

**DOI:** 10.1007/s00330-026-12334-9

**Published:** 2026-01-30

**Authors:** Maurizio Balbi, Luisella Righi, Noemi Cristina Culasso, Serena Capelli, Anna Caroli, Giorgia Andrea Impalà, Paola Berchialla, Ludwig Federico Garello, Rouslan Senkeev, Davide Morbidelli, Giulia Reboli, Stefano Levra, Francesco Passiglia, Nicola Sverzellati, Andrea Veltri

**Affiliations:** 1 Radiology Unit, San Luigi Gonzaga Hospital, Department of Oncology, University of Turin, Orbassano, Italy; 2 Pathology Unit, San Luigi Gonzaga Hospital, Department of Oncology, University of Turin, Orbassano, Italy; 3 Bioengineering Department, Istituto di Ricerche Farmacologiche Mario Negri IRCCS, Ranica, Italy; 4 Pathology Unit, City of Health and Science, Department of Medical Sciences, University of Turin, Torino, Italy; 5 Department of Clinical and Biological Sciences, University of Turin, Orbassano, Italy; 6 Oncology Unit, San Luigi Gonzaga Hospital, Department of Oncology, University of Turin, Orbassano, Italy; 7 Scienze Radiologiche Unit, University Hospital of Parma, Department of Medicine and Surgery, University of Parma, Parma, Italy

**Keywords:** Biopsy, Lung diseases, Tomography, X-ray Computed, Case–Control studies

## Abstract

**Objectives:**

To assess the safety and diagnostic performance of CT-guided core needle biopsy (CNB) of focal pulmonary lesions with coexisting interstitial lung abnormalities (ILAs).

**Materials and methods:**

This retrospective 1:1 matched case–control study included patients with ILAs and controls who underwent CT-guided CNB of a focal pulmonary lesion from February 2010 to December 2023. Complications, nondiagnostic specimens, and CNB diagnostic performance for malignancy were compared. Logistic regression was used to identify predictors of complications and nondiagnostic specimens. Resected cases were reviewed for histopathological changes in the nonneoplastic lung.

**Results:**

Seventy-three patients with ILAs and matched controls were included (both groups: median age, 73.0 years; 13 women). No significant difference was found in complications (overall: 21/73 [29%] vs 24/73 [33%], *p* = 0.72; major: 2/73 [3%] vs 3/73 [4%], *p* = 1.00; minor: 19/73 [26%] vs 21/73 [29%], *p* = 0.85), nondiagnostic specimens (12/73 [16%] vs 9/73 [12%], *p* = 0.20), or diagnostic performance (accuracy: 89% [65/73] vs 96% [70/73], *p* = 0.11; sensitivity: 88% [61/69] vs 95% [62/65], *p* = 0.13; specificity: 100% [4/4] vs 100% [8/8], *p* = 1.00). Needle traversal of ILAs (OR, 7.04; 95% CI: 2.07–26.28; *p* = 0.008) and multiple pleural passes (OR, 8.06; 95% CI: 1.26–70.46; *p* = 0.03) were associated with complications. Nonneoplastic lung in ILAs revealed more complex histology and increased fibrotic features than controls.

**Conclusion:**

CT-guided CNB of focal pulmonary lesions with coexisting ILAs was as safe and accurate as in patients without ILAs. However, traversing ILAs and multiple pleural passes increased complication risk.

**Key Points:**

***Question*** *Whether CT-guided CNB of focal pulmonary lesions with coexisting ILAs is as safe and accurate as in those without ILAs*.

***Findings*** *CNB in patients with ILAs showed similar safety and diagnostic performance to controls; however, complications were more frequent when ILAs were traversed or multiple pleural passes were performed*.

***Clinical relevance*** *CNB is a safe and effective diagnostic tool in patients with ILAs. Avoiding ILA traversal and multiple pleural passes may help minimize the risk of complications*.

**Graphical Abstract:**

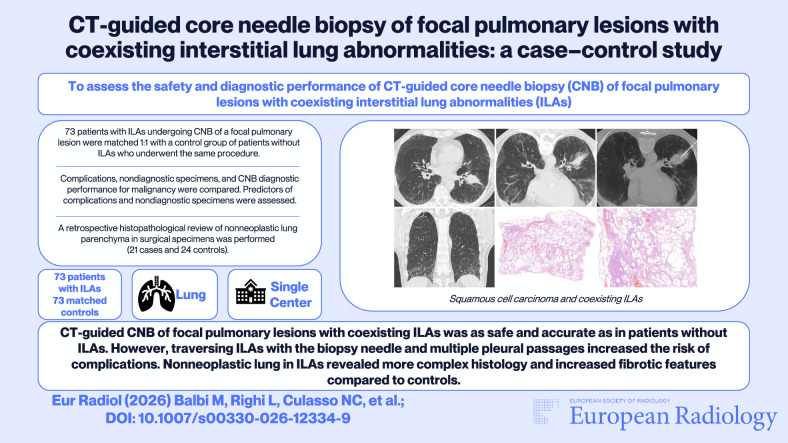

## Introduction

Interstitial lung abnormalities (ILAs) are incidental findings detected on partial or complete CT examination of the lungs that potentially reflect unsuspected interstitial lung disease (ILD) [1]. Although variably reported across study populations (e.g., prevalence rate: 1.2–16.7%) [1–3], ILAs are increasingly recognized owing to awareness of their potential clinical implications [4]. ILA-associated adverse outcomes include radiological progression, reduced lung function, respiratory disease, and a higher risk of lung cancer and lung cancer-related mortality [1, 2, 5–8]. The detection of a suspicious or indeterminate pulmonary lesion associated with ILAs warrants caution and a thorough workup to avoid missed or delayed lung cancer diagnosis.

CT-guided transthoracic needle biopsy (TTNB) is integral to the assessment of suspected lung cancer, especially for peripheral lesions, with > 90% success rates for obtaining adequate histopathologic analyses [9, 10]. TTNB can be performed with fine needle aspiration biopsy (FNAB), core needle biopsy (CNB), or a combination of both. Compared to FNAB, CNB is frequently preferred, as it allows the collection of larger amounts of tissue with acceptable rates of major complications (e.g., 4–6%) [11, 12]. Nonetheless, to the best of our knowledge, data on the safety and diagnostic performance of CT-guided CNB in ILAs are limited.

We hypothesized that ILAs may increase the risk of CNB-related complications, thereby potentially compromising patient safety and tissue-sampling adequacy. Indirect evidence to support this hypothesis derives from the established association between ILAs and complications of various lung cancer treatments, including surgery, radiotherapy, and immunotherapy [13–15]. Additionally, previous studies have reported increased risk of TTNB-related complications in patients with fibrotic ILD [16]. Whether this risk extends to ILAs remains unclear, as ILAs typically exhibit less advanced radiological involvement, including, but not limited to, the imaging hallmarks of pulmonary fibrosis. The potential impact of ILAs on the diagnostic performance of TTNB for pulmonary malignancies is still to be investigated.

This study aimed to evaluate the safety and diagnostic performance of CT-guided CNB of focal pulmonary lesions in patients with ILAs.

## Materials and methods

The IRB (Comitato Etico Territoriale Interaziendale, AOU Città della Salute e della Scienza di Torino) approved this retrospective observational case–control study and waived the requirement for written informed consent.

### Study population

The Picture Archiving and Communication System was searched for all patients who underwent CT-guided TTNB at the San Luigi Gonzaga Hospital (Orbassano, Turin) from February 2010 to December 2023. Records of identified patients, including CT images, were reviewed, in consensus, by a thoracic radiologist with 4 years of post-training experience (M.B.) and a 4th-year radiology resident (N.C.C.) to identify patients with ILAs who underwent CNB of a focal pulmonary lesion. ILAs were defined according to the 2020 Fleischner Society Position Paper as nondependent imaging findings that affect at least 5% of a lung zone (upper, middle, and lower lung zones were demarcated by the levels of the inferior aortic arch and right inferior pulmonary vein), including ground-glass or reticular abnormalities, parenchymal distortion, nonemphysematous cysts, honeycombing, and traction bronchiectasis [1]. The investigators assessed ILAs as a binary outcome (present vs absent) on the nearest CT scan performed within 3 months of CNB or, if this was unavailable, on the post-procedural whole-chest CT scan (technical details of the CT scans used to assess ILAs are reported in the Supplementary Material). Patients with a history of ILD or incomplete clinical data were excluded.

A control group of consecutive patients without ILAs was identified from the same set of patients screened by the initial search, at a 1:1 ratio to the final number of patients with ILAs. Control patients were matched by age ( ± 5 years), sex, lesion depth (± 5 mm), lesion dimension (± 5 mm), and emphysema (at most, one category difference), assessing these features consistent with a previous analysis [17], as described further in the “Methods” section.

### CNB protocol

CNBs were performed using a 6- or 64-detector-row CT scanner (Brilliance 6 or Ingenuity 64, respectively; Philips Medical Systems, Best, Netherlands), under sterile conditions and local anesthesia. A 17-gauge coaxial introducer (5, 10, or 15 cm in length) paired with an 18-gauge semi-automatic Tru-Cut needle was used to obtain one to four samples as appropriate [18]. Further details on the CNB protocol are provided in the Supplementary Material.

### Data collection

The previously noted group of investigators reviewed, in consensus, patients’ records to assess smoking history (non-smoker, past smoker, or current smoker), emphysema severity (absent, trace, mild, moderate, confluent, or advanced destructive) [19], CT features of ILAs, CT features of biopsied lesions, and procedural information.

Using the same CT scan as for patient selection, ILAs were classified as non-subpleural, subpleural non-fibrotic, and subpleural fibrotic, according to the 2020 Fleischner Society Position Paper [1]. The CT patterns of ILAs were also classified as typical usual interstitial pneumonia (UIP), probable UIP, indeterminate for UIP, or most consistent with a non-idiopathic pulmonary fibrosis (non-IPF) diagnosis [20]. The percentage of lung involvement related to ILAs was visually estimated to the nearest 5%.

The CT features of biopsied lesions and procedural information were assessed according to previous studies [17, 21–23] and are detailed in the Supplementary Material.

### Complications

Complications were extracted from the records and classified as minor or major, consistent with guidelines and previous literature [24–26]. Further details are provided in the Supplementary Material.

### Pathology review of biopsy reports and resection specimens

A thoracic pathologist with 13 years of post-training experience (L.R.), blinded to CT findings, reviewed the biopsy pathology reports and classified each biopsy result into one of the following five prespecified categories: malignancy, specific benignity, nonspecific benignity, atypical cells, or insufficient specimen [9, 16]. Results were then classified as diagnostic (i.e., malignancy or specific benignity) or nondiagnostic (i.e., nonspecific benignity, atypical cells, or insufficient specimen) [27]. In both groups, the same pathologist and a 4th-year pathology resident (G.A.I.) reviewed the available histologic slides for cases that underwent surgical resection to assess abnormalities in the nonneoplastic lung parenchyma [28].

### Determination of final diagnoses of biopsied lesions

The radiologist (M.B.) and pathologist (L.R.) jointly reviewed all available records, including surgical information and follow-up, to establish the final diagnoses of biopsied lesions. The diagnostic criteria were based on prior literature [16, 17, 29] and are summarized in Table S1. Based on the final diagnosis, each biopsied lesion was classified as either benign or malignant.

#### Statistical analysis

Patients with and without ILAs were compared for complication rates, proportion of nondiagnostic specimens, and diagnostic performance of CNB. The diagnostic performance of CNB was assessed in terms of accuracy, sensitivity, and specificity for detecting malignancy, using the final diagnosis as the reference standard. For these calculations, all CNB results other than malignancy (including nondiagnostic biopsies) were considered negative, consistent with previous studies [17, 30].

Age was dichotomized as < 65 years vs ≥ 65 years [17, 31], while Otsu’s method [32] was applied to other continuous variables.

In ILA patients, univariate and multivariate logistic regression analyses were performed to identify predictors of nondiagnostic specimens and complications. Variables with *p* < 0.10 in univariable analysis were tested in a stepwise multivariable model guided by the Akaike information criterion to identify the most relevant predictors for the final model. CNBs with > 1 pleural pass were excluded from nondiagnostic risk analyses due to uncertainty about which sample yielded the diagnosis.

Comparisons of continuous variables used the independent samples t-test for normal distributions, Welch’s t-test when variances were unequal, and the Mann–Whitney *U*-test for non-normal data. Categorical variables were analyzed with chi-square or Fisher’s exact test, depending on expected frequencies (Cochran’s rule). Pairwise comparisons used the Wilcoxon rank-sum test or Fisher’s exact test due to sample size imbalance or non-normality.

Conditional logistic regression analysis assessed whether ILAs increased complication risk or affected diagnostic yield, accounting for the matching variables.

Statistical significance was set at *p* < 0.05. All statistical analyses were performed using R software (R Core Team; https://www.r-project.org, version 4.4.1).

## Results

### Patient population and imaging characteristics of biopsied lesions and ILAs

**Fig. 1:**
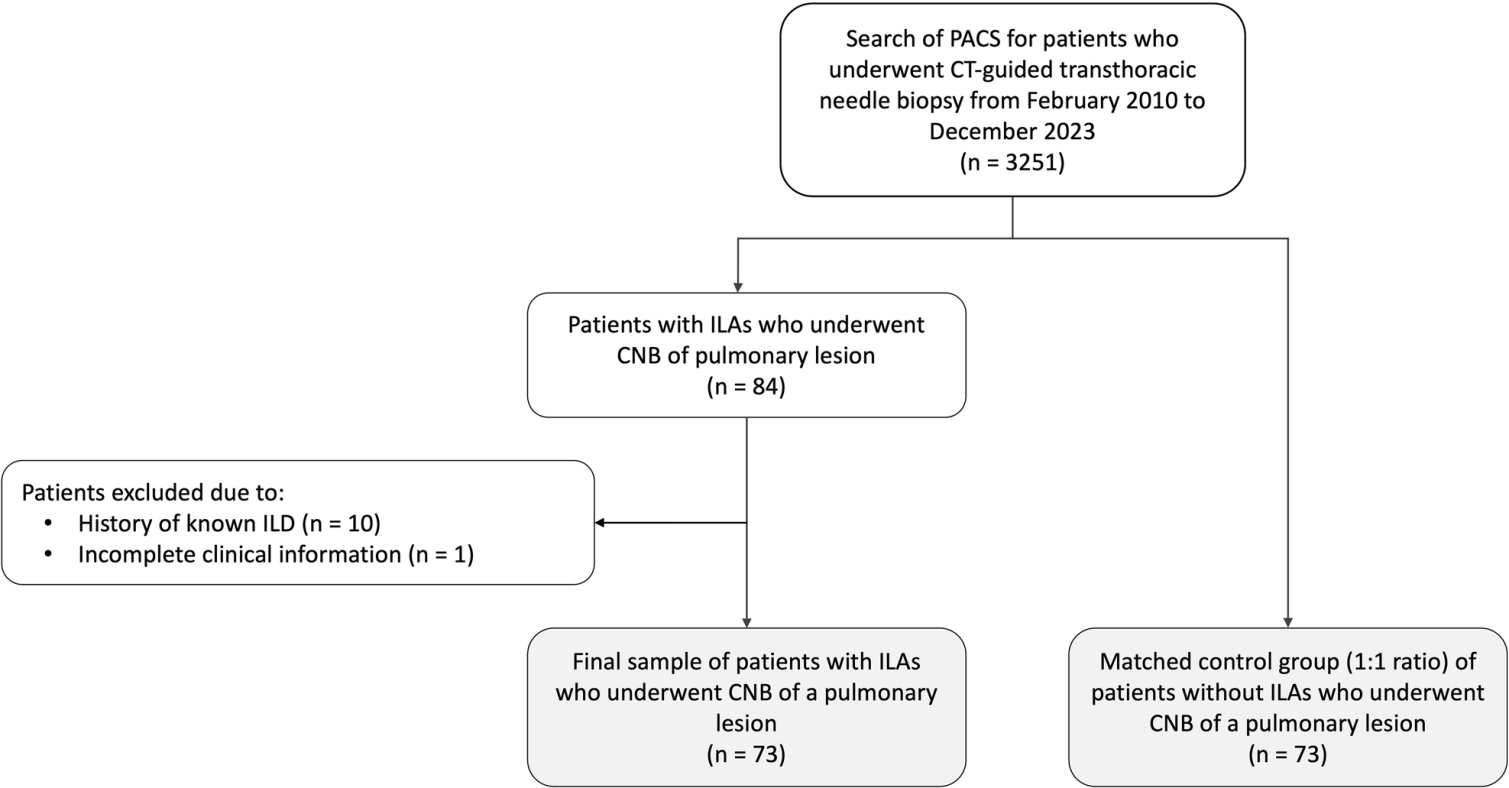
Flowchart of the patient selection process. Gray boxes indicate final study groups. CNB, core needle biopsy; ILAs, interstitial lung abnormalities; ILD, interstitial lung disease; PACS, picture archiving and communication system

Of 3251 patients who underwent CT-guided TTNB during the study period, 73 (2%) displayed ILAs. A matched control group of 73 patients who underwent the same procedure without radiological evidence of ILAs was then selected. Figure 1 shows the patient selection process. Demographics, emphysema severity, lesion dimension, and lesion depth of patients of both groups are reported in Table 1.

**Table 1:** Comparison of patients with ILAs (cases) and without ILAs (controls) in terms of variables used for matching, CNB results, and complications

	Cases	Controls	*p*
No.	73	73	
Patient age, years	73.0 [67.0–76.0]	73.0 [67.0–76.0]	1.00
Patient sex, women	13/73 (18%)	13/73 (18%)	1.00
Lesion dimension, mm^**^	30.8 [18.8–45.2]	31.5 [19.1–44.0]	0.50
Lesion depth, mm^**^	0.0 [0.0–0.0]	0.0 [0.0–0.0]	0.78
Emphysema severity^a^			0.99
Absent	24/73 (33%)	23/73 (32%)	
Trace	15/73 (21%)	17/73 (23%)	
Mild	12/73 (16%)	13/73 (18%)	
Moderate	13/73 (18%)	12/73 (16%)	
Confluent	2/73 (3%)	3/73 (4%)	
Advanced destructive	7/73 (10%)	5/73 (7%)	
Complication^**^			
Pneumothorax	13/73 (18%)	17/73 (23%)	0.54
Requiring chest-tube	2/73 (3%)	3/73 (4%)	1.00
Any bleeding^b^	11/73 (15%)	10/73 (14%)	1.00
Hemoptysis	1/73 (0%)	0/73 (0%)	1.00
Hemothorax	2/73 (3%)	4/73 (5%)	0.68
Parenchymal hemorrhage	11/73 (15%)	10/73 (14%)	1.00
Major bleeding	0/73 (0%)	0/73 (0%)	1.00
CNB results			0.40
Malignancy	61/73 (84%)	62/73 (85%)	
Specific benignity	0/73 (0%)	2/73 (3%)	
Nondiagnostic specimen	12/73 (16%)	9/73 (12%)	0.20
Nonspecific benignity	4/73 (5%)	7/73 (10%)	
Atypical cells	5/73 (7%)	2/73 (3%)	
Insufficient specimen	3/73 (4%)	0/73 (0%)	

Data are shown as median [IQR] or number/total (%)

*CNB* core needle biopsy, *ILAs* interstitial lung abnormalities

^a^ Graded according to the Fleischner Society [19]

^b^ The sum for types of bleeding is larger than the value for any bleeding because some patients experienced multiple forms of bleeding

^**^ Definitions are detailed in the Supplementary Material

Most coexisting ILAs involved ≤ 10% of lung parenchyma (*n* = 44/73, 60%). The majority of ILAs were either subpleural fibrotic (*n* = 49/73, 67%) or subpleural non-fibrotic (*n* = 21/73, 29%) (Table S2). The prevalence of typical UIP, probable UIP, indeterminate for UIP, and suggestive of non-IPF diagnosis CT patterns was 19% (*n* = 14/73), 32% (*n* = 23/73), 33% (*n* = 24/73), and 16% (*n* = 12/73), respectively. Subpleural fibrotic ILAs were characterized by traction bronchiectasis with honeycombing (*n* = 14/49, 29%) or without honeycombing (*n* = 35/49, 71%). Subpleural non-fibrotic ILAs were characterized by predominantly peripheral reticular opacities, sometimes with superimposed ground-glass opacities (*n* = 8/21, 38%). Non-subpleural ILAs (*n* = 3/73, 4%) showed peribronchovascular ground-glass and reticular abnormalities without fibrotic features.

### Complications

Patients with ILAs who underwent CNB, compared to those without ILAs undergoing the same procedure, showed no significant difference in the overall complication rate (*n* = 21/73 [29%] vs 24/73 [33%]; *p* = 0.72), frequency of major complications (regardless of concomitant minor complications) (*n* = 2/73 [3%] vs 3/73 [4%]; *p* = 1.00), or occurrence of minor complication only (*n* = 19/73 [26%] vs 21/73 [29%]; *p* = 0.85). Differences in individual complications were not significant between the two groups (Table 1).

CNB in patients with ILAs, compared to CNB in patients without ILAs, was not identified as a significant risk factor for complications (OR, 0.873 [95% CI: 0.418–1.823]; *p* = 0.72).

Variables that differed significantly between patients with and without complications included the number of pleural passes (*p* = 0.006), needle trespassing emphysema (*p* = 0.005), needle trespassing ILAs (*p* = 0.001), and needle dwell time (*p* = 0.03) (Table S2).

In the final multivariable model (Table 2), complication risk showed significant independent associations with needle trespassing ILAs (OR, 7.04 [95% CI: 2.07–26.28]; *p* = 0.008) and the number of pleural passes (OR, 8.06 [95% CI: 1.26–70.46]; *p* = 0.03). Needle dwell time was retained in the final multivariable model, but did not independently predict complication occurrence (OR, 3.04 [95% CI: 0.72–13.02]; *p* = 0.22).

**Table 2:** Predictive value of patient, procedure, and lesion data for CNB complications (left) and nondiagnostic specimens (right) in patients with ILAs

	Complications (*n* = 73)				Nondiagnostic specimens (*n* = 65)			
	Univariate LR		Multivariate LR		Univariate LR		Multivariate LR	
	OR (95% CI)	*p*	OR (95% CI)	*p*	OR (95% CI)	*p*	OR (95% CI)	*p*
Patient data								
Age, ≥ 65 years	1.26 (0.33–6.15)	0.75			0.46 (0.11–2.45)	0.32		
Sex, males	0.58 (0.17–2.16)	0.40			0.46 (0.11–2.45)	0.32		
Smoking history^*^								
Past smoker	0.61 (0.05–14.25)	0.70			NA	0.10		
Current smoker	1.20 (0.08–32.79)	0.90			NA	0.99		
Emphysema, yes	0.97 (0.34–2.97)	0.96			0.74 (0.19–3.13)	0.66		
ILAs subcategory^§^								
Subpleural non-fibrotic	0.25 (0.01–3.05)	0.29			NA	0.99		
Subpleural fibrotic	0.16 (0.01–1.84)	0.15			NA	0.10		
UIP diagnostic categories^#^								
Probable UIP	0.39 (0.11–1.23)	0.12			1.08 (0.05–5.50)	0.92		
Typical UIP	0.11 (0.01–0.63)	**0.04**			2.60 (0.53–1.07)	0.23		
ILAs extent, ≥ 15%^°°^	0.50 (0.16–1.46)	0.22			1.18 (0.23–4.80)	0.82		
Procedure data								
No. of pleural passes, > 1	0.10 (0.01–0.48)	**0.008**	8.06 (1.26–70.46)	**0.03**	---	---		
Needle trespassing lung parenchyma, yes	3.20 (0.13–9.75)	**0.03**			0.45 (0.12–1.69)	0.24		
Patient position, supine or prone	0.75 (0.24–2.48)	0.62			1.43 (0.32–10.14)	0.67		
Needle trespassing fissure, yes	NA	NA			NA	NA		
Needle trespassing emphysema, yes	NA	0.99			11.78 (1.03–269.73)	**0.053**	11.78 (1.03–269.73)	0.053
Needle trespassing ILAs, yes	7.33 (2.40–24.22)	**< 0.001**	7.04 (2.07–26.28)	**0.008**	1.18 (0.23–4.80)	0.82		
Pleural angle, ≥ 30^°,**^	1.24 (0.44–3.45)	0.68			1.03 (0.99–1.06)	0.34		
Needle angle with respect to gravity, closer to vertical^**^	0.81 (0.28 – 2.50)	0.71			2.07 (0.47 – 14.53)	0.38		
Needle dwell time, ≥ 17 min^**^	3.96 (1.21–13.40)	**0.02**	3.04 (0.72–13.02)	0.22	1.31 (0.26–5.38)	0.72		
Lesion data								
Lung lobe, lower lobes	0.78 (0.28–2.16)	0.63			1.75 (0.47–7.33)	0.41		
Depth, ≥ 20 mm^**^	5.37 (0.49–119.52)	0.18			NA	0.99		
Dimension, < 42 mm^**^	0.48 (0.12–1.55)	0.25			1.81 (0.47–6.86)	0.38		

The first column of the table presents categorical variables along with their respective levels, separated by a comma. For binary variables (e.g., ILAs extent), one level is explicitly mentioned (e.g., ≥ 15%), while for variables with more than two levels (e.g., ILAs subcategory), all levels are listed except for the reference level (e.g., subpleural non-fibrotic, subpleural fibrotic). Odds ratios, 95% CI, and *p*-values were computed by univariate ordinal logistic regression models. Variables found to be significant in the univariate analysis (*p* < 0.10, in bold) were included in the multivariate ordinal logistic model, which was further reduced using the AIC stepwise model selection technique. Variables found to be significant in the final multivariate model (*p* < 0.05) are indicated in bold. NA indicates that the calculation was not feasible due to low event counts and an imbalanced distribution of patients across subgroups, leading to numerical instability

*AIC* Akaike’s information criterion, *CNB* core needle biopsy, *ILAs* interstitial lung abnormalities, *LR* logistic regression, *UIP* usual interstitial pneumonia

^*^Thirty-two missing values

^§^According to the Fleischner Society [1]

^#^Based on CT appearance, according to the Fleischner Society [20]

^°°^Refers to visual estimation on CT, rounded to 5%

^**^Definitions are detailed in the Supplementary Material

### Nondiagnostic specimens

CNB showed malignancy in 84% (*n* = 61/73) and nondiagnostic specimens in 16% (*n* = 12/73) of pulmonary lesions with coexisting ILAs (Table 1). The frequency of a nondiagnostic specimen did not significantly differ between cases and controls (*n* = 12/73 [16%] vs 9/73 [12%]; *p* = 0.20).

CNB in patients with ILAs, compared to those without ILAs, was not a significant risk factor for a nondiagnostic specimen (OR, 1.46 [95% CI: 0.55–3.90]; *p* = 0.45).

In the 65 patients whose lesions were biopsied by a single pleural pass, those with diagnostic and nondiagnostic results showed no significant difference for any procedural-, patient-, or lesion-related factor (Table S2). In multivariable analysis, no factor was independently associated with the risk of a nondiagnostic specimen (Table 2).

### Histopathological analyses

Final diagnoses of pulmonary lesions in patients with and without ILAs are summarized in Table S3. Malignancy was found in 95% (*n* = 69/73) of patients with ILAs and 89% (*n* = 65/73) of those without ILAs. In both groups, the most common malignancy was adenocarcinoma (*n* = 27/69 [39%] in cases; 38/65 [58%] in controls), followed by squamous cell carcinoma (*n* = 23/69 [33%] in cases; 15/65 [23%] in controls).

Pulmonary lesions were resected in 29% (*n* = 21/73) of patients with ILAs and in 33% (*n* = 24/73) of patients without ILAs, with histologic slides available for review.

Retrospective review of the nonneoplastic lung parenchyma revealed that patients with radiological evidence of ILAs exhibited a more complex histologic pattern compared to controls, with higher rates of fibroblastic foci (*p* < 0.001) and honeycombing (*p* < 0.001) and an increased frequency of fibrotic features, such as subpleural fibrosis (*p* < 0.001) and patchy interstitial fibrosis (*p* < 0.001) (Table 3). Histologic findings in nonneoplastic lung parenchyma, stratified by the diagnostic categories of UIP based on CT appearance, are reported in Table S4.

**Table 3:** Histopathologic features of nonneoplastic lung parenchyma of patients with ILAs (cases) and without ILAs (controls)

	Cases (*n* = 21)	Controls (*n* = 24)	*p*
Fibrosis^a^			
Patchy interstitial fibrosis	10/21 (48%)	0/24 (0%)	**< 0.001**
Subpleural fibrosis	17/21 (81%)	0/24 (0%)	**< 0.001**
Peribronchiolar fibrosis	3/21 (14%)	8/24 (33%)	0.14
Diffuse interstitial thickening	4/21 (19%)	4/24 (16%)	1.00
Emphysematous fibrosis	4/21 (19%)	5/24 (21%)	1.00
Dense fibrosclerosis	6/21 (29%)	0/24 (0%)	**0.007**
Smoking-related interstitial fibrosis	9/21 (43%)	0/24 (0%)	**< 0.001**
Normal parenchyma	0/21 (0%)	4/24 (16%)	0.11
Additional histopathologic features^a^			
Fibroblastic foci	11/21 (52%)	1/24 (4%)	**< 0.001**
Honeycombing	12/21 (57%)	0/24 (0%)	**< 0.001**
Organizing pneumonia	4/21 (19%)	1/24 (4%)	0.17
Desquamative interstitial pneumonia	1/21 (5%)	1/24 (4%)	1.00
Respiratory bronchiolitis	2/21 (10%)	0/24 (0%)	0.21
Emphysema	3/21 (14%)	6/24 (25%)	0.47
Bronchiectasis	2/21 (10%)	0/24 (0%)	0.21
Anthracosis	8/21 (38%)	4/24 (16%)	0.11

*p*-values were computed using the Fisher exact test or chi-square test, according to the expected frequencies (Cochran's rule). Significant differences are highlighted in boldface

*ILAs* interstitial lung abnormalities

^a^ In patients with ILAs, the combined count of fibrotic and additional histopathologic features exceeds the total number of histopathologic findings, as patients exhibited multiple concurrent abnormalities

**Fig. 2:**
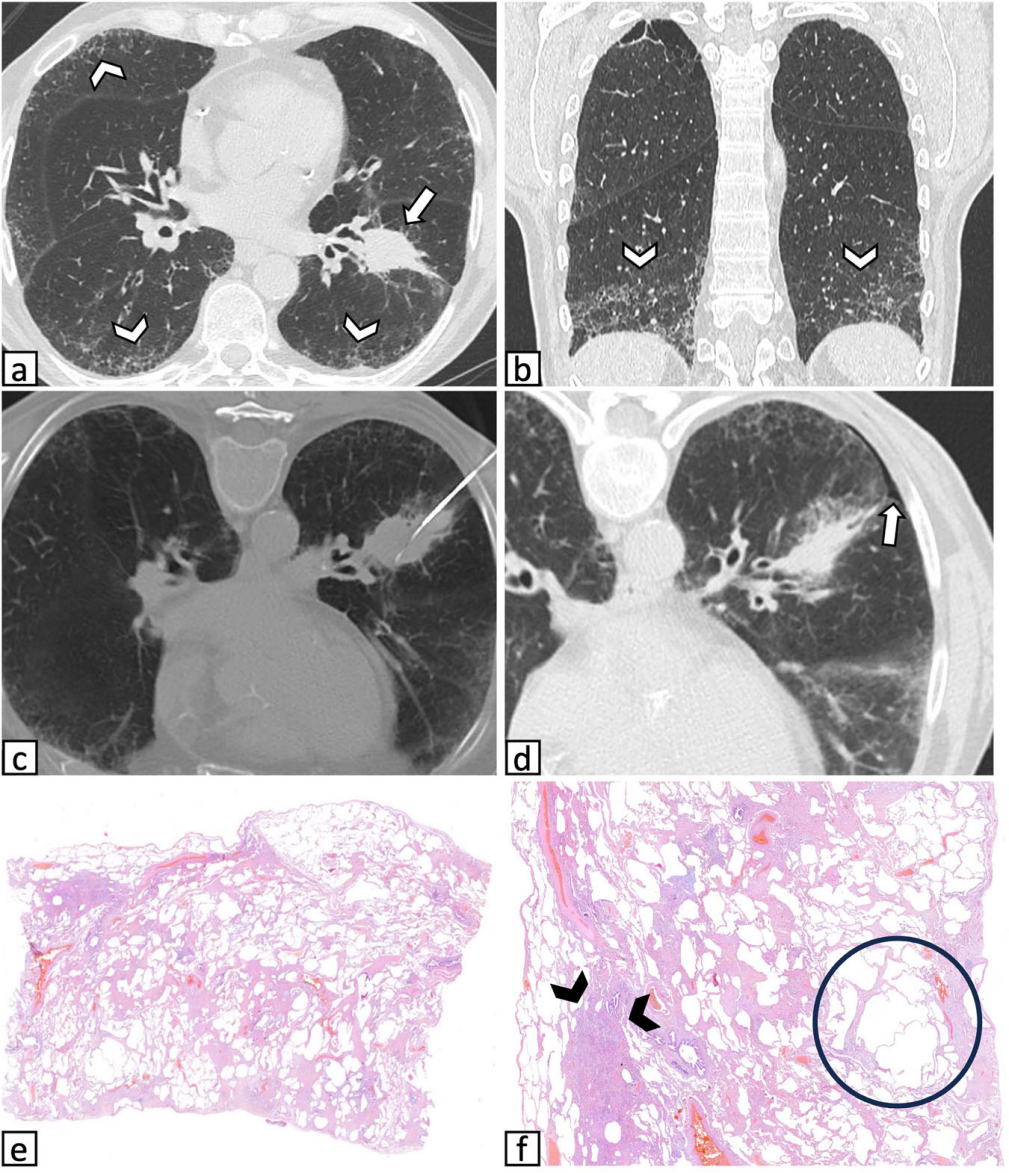
Representative images of a 70-year-old man with ILAs who underwent CT-guided CNB of a pulmonary mass: **a** axial CT image acquired before needle insertion shows a solid mass located in the left lower lobe (arrow) with coexisting subpleural non-fibrotic ILAs (arrowheads) predominantly characterized by reticulations admixed with emphysematous changes. **b** Coronal CT reconstruction shows lower lobe predominance of ILAs (arrowheads). **c** The lesion was targeted by positioning the patient prone, and the final diagnosis was squamous cell carcinoma. **d** A small pneumothorax was identified in the postprocedural CT acquisition (arrow), not requiring treatment. **e**, **f** Histological images of the nonneoplastic resected parenchyma. **e** At lower magnification, diffuse non-specific fibrotic changes are observed. **f** At higher magnification, dense fibrosclerosis (arrowheads) is alternated with emphysematous fibrosis (circle), suggesting smoking-related damage. CNB, core needle biopsy; ILAs, interstitial lung abnormalities

**Fig. 3:**
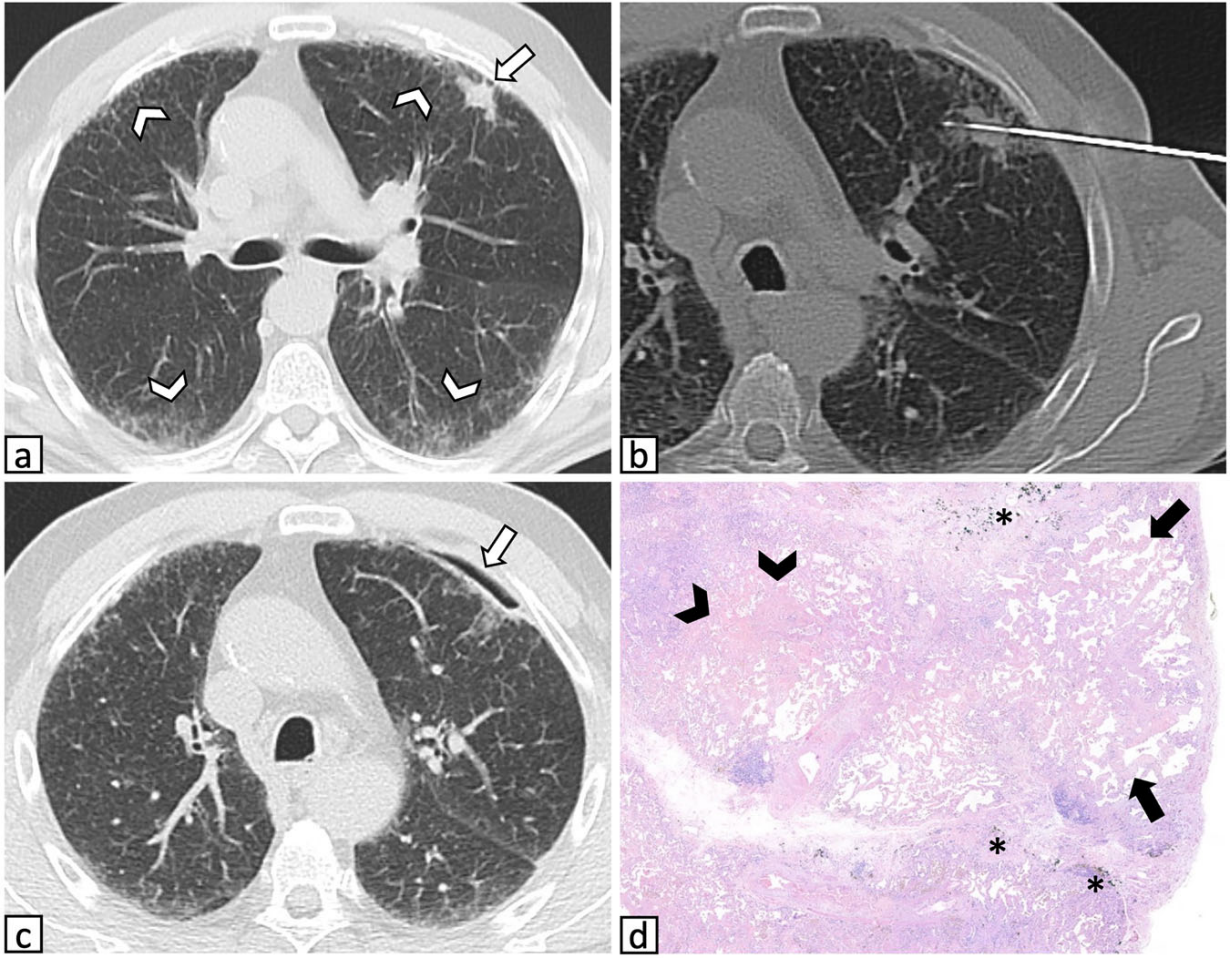
Representative images of a 72-year-old man who underwent CT-guided CNB of a pulmonary nodule in pronounced ILAs: **a** axial CT image obtained prior to needle insertion shows a suspicious solid nodule located in the left upper lobe (arrow) with coexisting subpleural non-fibrotic ILAs characterized by fine reticulations (arrowheads). **b** Procedural axial CT image shows the needle targeting the lesion while traversing ILAs. The final diagnosis was adenocarcinoma. **c** A small pneumothorax was identified in the postprocedural CT acquisition but did not require treatment (arrow). **d** Histological image of the peritumoral resected parenchyma at low magnification shows diffuse interstitial thickening (arrows), along with anthracosis (asterisks) and small areas of dense fibrosclerosis (arrowheads), suggesting smoking-related damage. CNB, core needle biopsy; ILAs, interstitial lung abnormalities

**Fig. 4:**
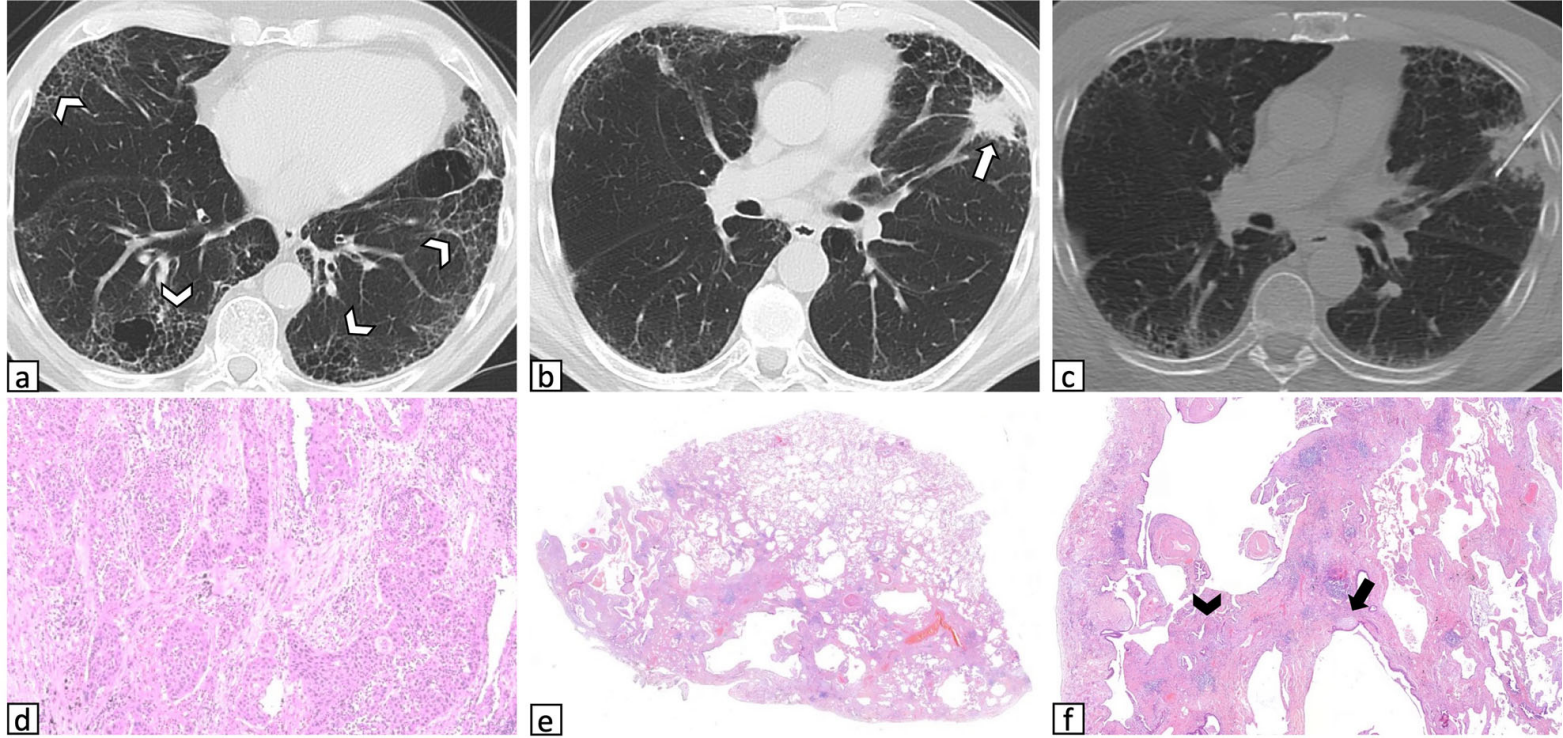
Representative images of a 70-year-old man with ILAs who underwent CT-guided CNB of a pulmonary mass: **a** CT image at lower lung zones depicts subpleural fibrotic ILAs characterized by bronchiectasis and architectural distortion, along with emphysematous changes and clustered subpleural cysts suggestive of honeycombing (arrowheads) **b** CT image acquired before needle insertion shows a suspicious solid mass located in the left upper lobe (arrow). **c** The procedure was performed with the patient positioned supine, and no postprocedural complications occurred. **d** Histological image of the resected biopsied lesion depicts squamous cell carcinoma. **e**, **f** Histological images of the peritumoral resected parenchyma. **e** At lower magnification, subpleural dense fibrotic areas are heterogeneously alternated with normal parenchyma. **f** At higher magnification, patchy scarring fibrosis with honeycombing changes (arrowhead) and fibroblastic foci (arrow) can be appreciated. CNB, core needle biopsy; ILAs, interstitial lung abnormalities

Figures 2– 4 show CT images of cases from the ILA group, along with the corresponding histology.

### Diagnostic performance

CNBs of lesions in patients with ILAs vs those without ILAs were associated with an accuracy of 89% (65/73) vs 96% (70/73) (*p* = 0.11), sensitivity of 88% (61/69) vs 95% (62/65) (*p* = 0.13), and specificity of 100% (4/4) vs 100% (8/8) (*p* = 1.00) for the diagnosis of malignancy, using the final diagnosis as the reference standard.

## Discussion

CNB is a well-established method for characterizing pulmonary lesions. However, limited data are available on its safety and diagnostic accuracy for diagnosing malignancy in patients with coexisting ILAs. Addressing this issue is of diagnostic and prognostic relevance because ILAs carry an increased risk of lung cancer while conferring a certain degree of lung fragility. This susceptibility to lung damage might impact the safety and the diagnostic accuracy of CNB, with relevant implications on patient prognosis.

This study found that CNB of focal pulmonary lesions in patients with CT imaging evidence of coexisting ILAs is safe and accurate, without significant differences in the frequency of minor or major complications, nondiagnostic results, or diagnostic performance for malignancy compared with CNB performed in patients without ILAs. Nonetheless, traversing ILAs with the biopsy needle increased the occurrence of complications. This was also true for performing > 1 pleural pass, whereas no factor was independently associated with a nondiagnostic specimen.

The results suggest that CNB constitutes a safe diagnostic option for assessing pulmonary lesions in patients with ILAs, offering a safety profile comparable to that observed in patients without such abnormalities. Indeed, the frequency of major and minor complications after CNB was similar between patients with and without concomitant ILAs and aligned with those of CT-guided TTNB in general [12]. Consistent with prior studies in patients with overt ILDs [16, 33, 34] and in larger unselected cohorts [12, 35], the most common biopsy complication was pneumothorax, usually without necessitating chest-tube placement, followed by pulmonary hemorrhage. Notably, despite the reported association between ILAs and an increased risk of acute respiratory distress syndrome [36], we did not record any case of acute respiratory deterioration triggered by CNB.

The diagnostic performance in patients with ILAs was slightly lower but remained comparable to the literature reporting accuracies of 90–95% [9, 17, 37]. Whether this trend toward reduced accuracy may reach statistical significance in larger cohorts has to be determined. However, no specific risk factors for nondiagnostic sampling were identified in the ILA group, which suggests the absence of a distinct mechanism of biopsy failure in this population. As the majority of ILAs in the present study were limited in extent, this assumption may not be generalizable to patients with more advanced parenchymal involvement, in whom factors such as reduced respiratory cooperation could interfere with lesion targeting and compromise sampling accuracy.

Needle traversal of ILAs emerged as an independent risk factor for complications, but the underlying mechanism remains uncertain. In a prior study, increased complication risk was significantly associated with needle traversal of honeycombing [16] —an established hallmark of pulmonary fibrosis—thereby supporting the hypothesis that changes in pulmonary elasticity may be a determinant of TTNB-related complications. Nevertheless, in our sample, most traversed ILAs consisted of reticulations without overt fibrotic features, and complication rates were similar across ILA subtypes, which suggests that factors other than pulmonary fibrosis may have predisposed our patients to needle-induced injury. Consistent with prior literature, complication rates also increased with the number of pleural punctures [38, 39]. Other reports have identified additional risk factors, such as small lesion size and deep lesion location [40–42], which, in practice, may sometimes lead to almost unavoidable complications. Possibly owing to the limited sample size, none of these predictors reached statistical significance in the present analysis.

Histopathologic examination of nonneoplastic lung tissue from patients with ILAs revealed a significantly higher proportion of multiple abnormalities compared to controls. Importantly, some of the histopathologic findings we identified in association with ILAs, such as fibroblastic foci, subpleural fibrosis, patchy interstitial fibrosis, and honeycombing, are conventional hallmarks to define a UIP pattern (i.e., the histopathologic correlate of IPF) [43]. These findings are similar to those reported by prior studies wherein some ILAs, evaluated in surgical specimens obtained during lung nodule or cancer resections, demonstrated histopathologic features of UIP [13, 28]. Although these cases may have reflected undiagnosed IPF or other forms of ILD [43, 44], evaluation of their progression and eventual diagnosis was beyond the scope of the present study. The clinical significance of incidental histologic interstitial changes detected in lung cancer resections remains debatable. To improve their understanding and support multidisciplinary evaluation, radiologists who plan TTNB should be encouraged to systematically assess and highlight the presence of ILAs, especially when previously underreported.

This study had some limitations. First, some of the risk factors for nondiagnostic results or complications may not have demonstrated statistical significance due to the small sample size. Second, uncollected variables (e.g., coagulation status, pulmonary hypertension) may have represented unmeasured confounders. Third, given that most of the targeted lesions were juxtapleural, our findings may not be generalizable to more centrally located lesions. Fourth, we could not reliably explore the impact of operator experience, a factor that affected safety and diagnostic accuracy in previous studies [42]. Fifth, assessment of ILAs may have been suboptimal in cases where only post-biopsy images were available, as superimposed procedure-related changes may have influenced their evaluation, and in cases where scans did not meet the criteria for HRCT (i.e., slice thickness ≤ 1.5 mm with a high-spatial-frequency reconstruction kernel). Sixth, investigators performed the data collection process in consensus; inter-reader agreement was not assessed for characteristics of ILAs. Seventh, ILAs were assessed according to the 2020 Fleischner Society Position Paper [1]; some cases included in the study might have been classified as ILD if the 2025 American Thoracic Society Clinical Statement had been applied [45], whereas some patients may have been included in the study population under American Thoracic Society but not Fleischner recommendations. Eight, in nondiagnostic risk analyses, exclusion of CNBs with > 1 pleural pass may have biased results toward technically easier procedures and led to underestimation of some predictors. Finally, because surgical procedures were not designed to provide information about ILAs, it is possible that the most informative histological samples with respect to ILAs were missed by gross sampling.

In conclusion, our findings suggest that CNB of focal pulmonary lesions in patients with coexisting ILAs can be performed with diagnostic accuracy and safety that are comparable to those observed in patients without ILAs. When technically feasible, limiting the procedure to a single pleural puncture and avoiding needle traversal of ILAs may represent a careful strategy to minimize procedural risks in this patient population.

## Supplementary information

Supplementary information

## Abbreviations

CNB: Core needle biopsy
FNAB: Fine needle aspiration biopsy
ILA: Interstitial lung abnormality
ILD: Interstitial lung disease
IPF: Idiopathic pulmonary fibrosis
TTNB: Transthoracic needle biopsy
UIP: Usual interstitial pneumonia
