# Supplementary material for: CT-guided core needle biopsy of focal pulmonary lesions with coexisting interstitial lung abnormalities: a case-control study
Source: Eur Radiol. 2026 Jan 30;36(6):4366–77. doi: 10.1007/s00330-026-12334-9 (PMC13212682; doi:10.1007/s00330-026-12334-9)
Supplement: Supplementary file 1 — Supplementary information [file 330_2026_12334_MOESM1_ESM.docx]

**Supplementary Material**

## *CNB procedure*

The patient position (i.e., prone, supine, oblique, or lateral decubitus) was chosen to ensure the shortest and safest needle pathway, avoiding emphysema, major vessels, and fissures whenever possible.

All procedural steps followed a “move off and scan” approach to minimize radiation exposure. Axial CT images of the area of interest were acquired to monitor the needle course up to the targeted lesion. A postprocedural whole chest CT was acquired to check for immediate complications using the lung window setting to detect pneumothorax or hemorrhage. No post-biopsy prophylactic measures, such as blood patching or rapid roll-over, were applied.

The recovery protocol consisted of 2 hours of continuous monitoring of vital signs followed by chest radiography. In the absence of supervening complications, patients were discharged on the same day of the procedure or, if inpatients, returned to their hospital room; otherwise, they were managed according to the standard of care.

*CT features of targeted lesions and procedural information*

CT features of targeted lesions included lesion lobe, depth (distance from the pleura to the lesion’s outer margin), consistency (solid, part-solid, ground-glass), and dimension (recorded as the mean of the maximal long axis and maximal perpendicular short axis in the axial plane).

Procedural information included the number of pleural passes, patient position (prone, supine, oblique, or lateral decubitus), needle traversal of lung parenchyma (yes vs. no), needle traversal of emphysema (yes vs. no), needle traversal of fissures (yes vs. no), needle traversal of ILAs (yes vs. no), needle traversal of honeycombing (yes vs. no), needle traversal of bronchiectasis (yes vs. no), needle traversal of reticulation (yes vs. no), pleural angle, needle angle to gravity, and needle dwell time. The pleural angle was computed as the deviation angle between the needle’s longitudinal axis and a line drawn perpendicular to the tangent to the pleura at the needle’s entry point, as illustrated previously [21]. The needle angle to gravity was calculated using the CT table as a horizontal frame of reference and classified as closer to vertical (>45° to 135°) or closer to horizontal (–45° to 45°) [22]. The needle dwell time was defined as the time that elapsed between pleural puncture and needle removal [23]. Factors assessed in patients without ILAs included emphysema severity, lesion depth, and lesion dimension.

*CT scans for ILA evaluation – technical details*

For the evaluation of ILAs, immediate post-procedural whole-chest scans were used in 38/73 (52%) cases. The remaining scans were acquired within a median time interval of 42 days (IQR, 17.8–66 days) before or after the procedure. All scans were performed without the use of a contrast medium, with the patient positioned in a prone (35/73, 48%), supine (30/73, 41%), or oblique/lateral (8/73, 11%) position. Parameters of procedural scans were as follows: tube voltage, 100–120 kVp; tube current, 68–206 mAs; pitch, 0.9–1.4; slice thickness, 1.5–5 mm; revolution time, 0.4–0.75 s; FOV, 346–500 mm; matrix, 512x512. Parameters of scans acquired outside the biopsy setting were as follows: 120 kVp; tube current, 70–352 mAs; pitch, 0.6–1.5; slice thickness, 1.25–4 mm; revolution time, 0.4–1.0 s; FOV, 313–474 mm; matrix, 512x512. Overall, HRCT criteria, defined as slice thickness ≤1.5 mm coupled with a high spatial reconstruction algorithm, were met in 11/73 (15%) cases.

*Complications*

Transient hemoptysis not requiring treatment, self-limiting hemothorax, pneumothorax without chest tube placement, and pulmonary hemorrhage >20 mm in width not requiring treatment were classified as minor complications, whereas pneumothorax with chest tube placement, bleeding requiring treatment, air embolism, and death were classified as major complications [24]. Hemorrhages measuring ≤20 mm in width along the needle tract and pneumothorax measuring ≤1 cm were considered normal postprocedural findings [25, 26].

**Table S1. Criteria for definite diagnosis**

| 1. Surgical pathological results were used for diagnosis when lesions were surgically resected. |  |
| --- | --- |
| 1. Nonsurgical biopsy results were accepted as final diagnoses when they showed malignant or specific benign pathologic findings. 2. If lesions decreased by 20% or more in diameter or remained stable in size for at least 2 years, they were considered benign. | |
| 1. If lesions showed an obvious malignant clinical course during the follow-up, they were considered malignant. |  |

**Table S2. Patient, procedure, and lesion data of 73 cases with ILAs grouped based on complication occurrence (left) and 65 cases with ILAs with a single needle pleural pass stratified based on diagnostic yield (right).**

|  | **Complications** | | | | **Diagnostic yield** | | | |
| --- | --- | --- | --- | --- | --- | --- | --- | --- |
|  | **All cases** | **With complications** | **Without complications** | ***p*** | **All cases** | **Diagnostic specimens** | **Nondiagnostic specimens** | ***p*** |
| No. | 73 | 21 | 52 |  | 65 | 54 | 11 |  |
| **Patient data** |  |  |  |  |  |  |  |  |
| Age, <65 years | 12/73 (16%) | 3/21 (14%) | 9/52 (17%) | 0.70 | 11/65 (17%) | 8/54 (15%) | 3/11 (27%) | 0.38 |
| Sex, women | 13/73 (18%) | 5/21 (24%) | 8/52 (15%) | 0.50 | 11/65 (17%) | 8/54 (15%) | 3/11 (27%) | 0.38 |
| Smoking history* |  |  |  | 0.61 |  |  |  | 0.55 |
| Non-smoker | 3/41 (7%) | 1/11 (9%) | 2/30 (7%) |  | 3/39 (8%) | 3/34 (9%) | 0/5 (0%) |  |
| Past smoker | 30/41 (73%) | 7/11 (64%) | 23/30 (77%) |  | 28/39 (72%) | 25/34 (74%) | 3/5 (60%) |  |
| Current smoker | 8/41 (20%) | 3/11 (27%) | 5/30 (17%) |  | 8/39 (21%) | 6/34 (18%) | 2/5 (40%) |  |
| Emphysema, yes | 49/73 (67%) | 14/21 (67%) | 35/52 (67%) | 1.00 | 45/65 (69%) | 38/54 (70%) | 7/11 (64%) | 0.73 |
| ILAs subcategory^§^ |  |  |  | 0.25 |  |  |  | 0.13 |
| Non-subpleural | 3/73 (4%) | 2/21 (10%) | 1/52 (2%) |  | 3/65 (5%) | 3/54 (6%) | 0/11 (0%) |  |
| Subpleural non-fibrotic | 21/73 (29%) | 7/21 (33%) | 14/52 (27%) |  | 18/65 (28%) | 12/54 (22%) | 6/11 (55%) |  |
| Subpleural fibrotic | 49/73 (67%) | 12/21 (57%) | 37/52 (71%) |  | 44/65 (68%) | 39/54 (72%) | 5/11 (45%) |  |
| UIP diagnostic categories^#^ |  |  |  | 0.06 |  |  |  | 0.60 |
| Suggestive of non-IPF diagnosis | 12/73 (16%) | 6/21 (29%) | 6/52 (12%) |  | 11/65 (17%) | 9/54 (17%) | 2/11 (18%) |  |
| Indeterminate for UIP | 24/73 (33%) | 9/21 (43%) | 15/52 (29%) |  | 19/65 (29%) | 17/54 (31%) | 2/11 (18%) |  |
| Probable UIP | 23/73 (32%) | 5/21 (24%) | 18/52 (35%) |  | 21/65 (32%) | 18/54 (33%) | 3/11 (27%) |  |
| Typical UIP | 14/73 (19%) | 1/21 (5%) | 13/52 (25%) |  | 14/65 (22%) | 10/54 (19%) | 4/11 (36%) |  |
| ILAs extent°° |  |  |  | 0.55 |  |  |  | 1.00 |
| 5% | 19/73 (26%) | 6/21 (29%) | 13/52 (25%) |  | 19/65 (29%) | 16/54 (30%) | 3/11 (27%) |  |
| 10% | 25/73 (34%) | 9/21 (43%) | 16/52 (31%) |  | 21/65 (32%) | 17/54 (31%) | 4/11 (36%) |  |
| 15% | 9/73 (12%) | 1/21 (5%) | 8/52 (15%) |  | 9/65 (14%) | 8/54 (15%) | 1/11 (9%) |  |
| 20% | 8/73 (11%) | 3/21 (14%) | 5/52 (10%) |  | 7/65 (11%) | 6/54 (11%) | 1/11 (9%) |  |
| >20% | 12/73 (16%) | 2/21 (10%) | 10/52 (19%) |  | 9/65 (14%) | 7/54 (13%) | 2/11 (18%) |  |
| **Procedure data** |  |  |  |  |  |  |  |  |
| No. of pleural passes, >1 | 8/73 (11%) | 6/21 (29%) | 2/52 (4%) | **0.006** | --- | --- | --- | --- |
| Needle trespassing lung parenchyma, yes | 34/73 (47%) | 14/21 (67%) | 20/52 (38%) | 0.054 | 28/65 (43%) | 24/54 (44%) | 4/11 (36%) | 0.75 |
| Patient position, supine or prone | 55/73 (75%) | 15/21 (71%) | 40/52 (77%) | 0.64 | 50/65 (77%) | 41/54 (76%) | 9/11 (82%) | 0.64 |
| Needle trespassing fissure, yes | 0/73 (0%) | 0/21 (0%) | 0/52 (0%) | NA | 0/65 (0%) | 0/54 (0%) | 0/11 (0%) | NA |
| Needle trespassing emphysema, yes | 4/73 (5%) | 4/21 (19%) | 0/52 (0%) | **0.005** | 3/65 (5%) | 1/54 (2%) | 2/11 (18%) | 0.07 |
| Needle trespassing ILAs, yes | 20/73 (27%) | 12/21 (57%) | 8/52 (15%) | **0.001** | 16/65 (25%) | 13/54 (24%) | 3/11 (27%) | 1.00 |
| Needle trespassing honeycombing, yes | 1/20 (5%) | 0/12 (0%) | 1/8 (13%) | 0.400 | 1/16 (6%) | 1/13 (8%) | 0/3 (0%) | 1.000 |
| Needle trespassing bronchiectasis, yes | 2/20 (10%) | 0/12 (0%) | 2/8 (25%) | 0.147 | 2/16 (13%) | 2/13 (15%) | 0/3 (0%) | 1.000 |
| Needle trespassing reticulation, yes^##^ | 17/20 (85%) | 12/12 (100%) | 5/8 (63%) | 0.067 | 13/16 (81%) | 10/13 (77%) | 3/3 (100%) | 1.000 |
| Pleural angle, ≥30°** | 32/73 (44%) | 10/21 (48%) | 22/52 (42%) | 0.88 | 27/65 (42%) | 21/54 (39%) | 6/11 (55%) | 0.50 |
| Needle angle to gravity, closer to vertical** | 51/73 (70%) | 14/21 (67%) | 37/52 (71%) | 0.92 | 46/65 (71%) | 37/54 (69%) | 9/11 (82%) | 0.49 |
| Needle dwell time, ≥17 min** | 15/73 (21%) | 8/21 (38%) | 7/52 (13%) | **0.03** | 15/65 (23%) | 12/54 (22%) | 3/11 (27%) | 0.71 |
| **Lesion data** |  |  |  |  |  |  |  |  |
| Lung lobe, lower lobes | 38/73 (52%) | 10/21 (48%) | 28/52 (54%) | 0.82 | 34/65 (52%) | 27/54 (50%) | 7/11 (64%) | 0.52 |
| Depth, ≥20 mm** | 3/73 (4%) | 2/21 (10%) | 1/52 (2%) | 0.20 | 2/65 (3%) | 2/54 (4%) | 0/11 (0%) | 1.00 |
| Dimension, <42 mm** | 52/73 (71%) | 17/21 (81%) | 35/52 (67%) | 0.38 | 43/65 (66%) | 37/54 (69%) | 6/11 (55%) | 0.49 |

Unless otherwise specified, data are reported as the number of patients/total (%). The first column presents categorical variables along with their respective levels. p-values were computed using the Fisher exact test or chi-square test, according to the expected frequencies (Cochran's rule). Significant differences are highlighted in boldface. *32 missing values. §According to the Fleischner Society [1]. ^#^Based on CT appearance, according to the Fleischner Society [20]. °°Refers to visual estimation on CT, rounded to 5%. ^##^Admixed with ground-glass opacity in 3/17 (18%) cases. **Definitions are detailed in the Supplementary Material. Abbreviations: ILAs, interstitial lung abnormalities; IPF, idiopathic pulmonary fibrosis; UIP, usual interstitial pneumonia.

**Table S3**. **Final diagnoses of biopsied pulmonary lesions in patients with ILAs and matched controls.**

|  | **Patients with ILAs** | **Controls** |
| --- | --- | --- |
| **Final Diagnosis** | **No. of Patients/Total (%)** | **No. of Patients/Total (%)** |
| **Malignancy** | 69/73 (95%) | 65/73 (89%) |
| Adenocarcinoma | 27/69 (39%) | 38/65 (58%) |
| Squamous cell carcinoma | 23/69 (33%) | 15/65 (23%) |
| Undifferentiated carcinoma | 4/69 (6%) | 3/65 (5%) |
| Metastasis from extrapulmonary tumor | 3/69 (4%) | 5/65 (8%) |
| Sarcomatoid carcinoma | 1/69 (1%) | 2/65 (3%) |
| Malignancy of unknown histology* | 3/69 (4%) | 0/65 (0%) |
| Atypical carcinoid | 1/69 (1%) | 0/65 (0%) |
| Pleomorphic carcinoma | 0/69 (0%) | 1/65 (2%) |
| Adenosquamous carcinoma | 2/69 (3%) | 1/65 (2%) |
| Small cell lung carcinoma | 5/69 (7%) | 0/65 (0%) |
| **Benign finding** | 4/73 (5%) | 8/73 (11%) |
| Silicotic nodule | 0/4 (0%) | 1/8 (13%) |
| Hamartochondroma | 0/4 (0%) | 1/8 (13%) |
| Nonspecific inflammatory process | 4/4 (100%) | 6/8 (75%) |

*Occurrence of metastases at follow-up.

Abbreviations: ILAs, interstitial lung abnormalities.

**Table S4**. **Histopathologic features of nonneoplastic lung parenchyma and corresponding diagnostic categories of UIP based on CT pattern* in n=21 patients with ILAs**

|  | **Typical UIP (n=3)** | **Probable UIP (n=5)** | **Indeterminate for UIP (n=8)** | | **Suggestive of non-IPF diagnosis (n=5)** | ***p*** |
| --- | --- | --- | --- | --- | --- | --- |
| **Fibrosis** |  |  |  |  | |  |
| Patchy interstitial fibrosis | 2/3 (67%) | 4/5 (80%) | 3/8 (38%) | 1/5 (20%) | | 0.31 |
| Subpleural fibrosis | 3/3 (100%) | 5/5 (100%) | 6/8 (75%) | 3/5 (60%) | | 0.51 |
| Peribronchiolar fibrosis | 0/3 (0%) | 1/5 (20%) | 2/8 (25%) | 0/5 (0%) | | 0.85 |
| Diffuse interstitial thickening | 0/3 (0%) | 0/5 (0%) | 2/8 (25%) | 2/5 (40%) | | 0.51 |
| Emphysematous fibrosis | 1/3 (33%) | 0/5 (0%) | 1/8 (13%) | 2/5 (40%) | | 0.32 |
| Dense fibrosclerosis | 0/3 (0%) | 2/5 (40%) | 3/8 (38%) | 1/5 (20%) | | 0.77 |
| Smoking-related interstitial fibrosis | 0/3 (0%) | 1/5 (20%) | 4/8 (50%) | 4/5 (80%) | | 0.15 |
| Normal parenchyma | 0/3 (0%) | 0/5 (0%) | 0/8 (0%) | 0/5 (0%) | | NA |
| **Additional histopathologic features** |  |  |  |  | |  |
| Fibroblastic foci | 3/3 (100%) | 3/5 (60%) | 4/8 (50%) | 1/5 (20%) | | 0.21 |
| Honeycombing | 3/3 (100%) | 4/5 (80%) | 4/8 (50%) | 1/5 (20%) | | 0.15 |
| Organizing pneumonia | 0/3 (0%) | 1/5 (20%) | 2/8 (25%) | 1/5 (20%) | | 1.00 |
| Desquamative interstitial pneumonia | 0/3 (0%) | 0/5 (0%) | 1/8 (13%) | 0/5 (0%) | | 1.00 |
| Respiratory bronchiolitis | 0/3 (0%) | 1/5 (20%) | 1/8 (13%) | 0/5 (0%) | | 1.00 |
| Emphysema | 0/3 (0%) | 1/5 (20%) | 1/8 (13%) | 1/5 (20%) | | 1.00 |
| Bronchiectasis | 0/3 (0%) | 0/5 (0%) | 1/8 (13%) | 1/5 (20%) | | 1.00 |
| Anthracosis | 0/3 (0%) | 1/5 (20%) | 3/8 (38%) | 4/5 (80%) | | 0.14 |

Data are shown as number/total (%).

The p-value refers to the comparison between Typical UIP + Probable UIP vs. Indeterminate for UIP + Suggestive of non-IPF diagnosis. Fisher’s exact test was used in all comparisons, according to Cochran’s rule.

*Defined according to the Fleischner Society [20]. Abbreviations: ILAs, interstitial lung abnormalities; IPF, idiopathic pulmonary fibrosis; UIP, usual interstitial pneumonia.
